# On BH3 Mimetics and Ca^2+^ Signaling

**DOI:** 10.1002/ddr.21405

**Published:** 2017-08-13

**Authors:** Pawel E. Ferdek, Monika A. Jakubowska

**Affiliations:** ^1^ Medical Research Council Group, Cardiff School of Biosciences Cardiff University Cardiff Wales CF10 3AX United Kingdom

**Keywords:** Bcl‐2, BH3 mimetics, calcium, cell signaling, clinical trials, protein–protein interaction

## Abstract

**Table:** 

Preclinical Research

BH3 mimetics are anticancer agents that reproduce the spatial arrangement of the BH3 domain of Bcl‐2 family proteins. Just like the BH3‐only proteins, these compounds bind to the hydrophobic cleft of the pro‐survival Bcl‐2 members such as Bcl‐2 or Bcl‐xL, and disrupt their heterodimerization with pro‐apoptotic Bax or Bak, sensitizing cells to chemotherapy. In recent years, it has become clear that Bcl‐2 family proteins are engaged in regulation of intracellular Ca^2+^ homeostasis, including Ca^2+^ release from the intracellular stores as well as Ca^2+^ fluxes across the plasma membrane. Given that BH3 mimetics shift the balance between the prosurvival and proapoptotic Bcl‐2 members, they might indirectly exert effects on intracellular Ca^2+^ signals. Indeed, it has been reported that some BH3 mimetics release Ca^2+^ from the intracellular stores causing Ca^2+^ overload in the cytosol. Therefore, the effects of any new BH3 mimetics on cellular Ca^2+^ homeostasis should be tested before these compounds progress to clinical trials. Drug Dev Res 78 : 313–318, 2017. © 2017 Wiley Periodicals, Inc.

## BACKGROUND

The evolutionary conserved Bcl‐2 (B‐cell lymphoma 2) protein family consists of about 18 members very well known for their role in the process of programmed cell death. Based on their structure and functions these proteins have been categorized into three groups: (1) the prosurvival members, such as Bcl‐2 itself, along with Bcl‐xL, Bcl‐w or Mcl‐1; (2) the proapoptotic proteins (Bax, Bak); (3) and a divergent class of the proapoptotic BH3‐only proteins, including Bim, Bid, Puma, Noxa, and others. Prosurvival Bcl‐2 proteins bear four BH (Bcl‐2 homology) domains and usually a transmembrane domain at the C‐terminus. Bax and Bak have three BH domains (BH1–BH3) but their helix α1 somewhat resembles the BH4 domain of Bcl‐xL [Suzuki et al., 2000]. And the BH3‐only proteins have a single BH3 domain [Chipuk and Green, 2008]. The BH3 domain is an amphipathic α‐helix, consisting of 9–16 amino acids with conserved residues of leucine (Leu) and aspartic acid (Asp) [Aouacheria et al., 2015], that is responsible for the interaction with the hydrophobic cleft formed by BH1–BH3 domains of the pro‐survival Bcl‐2 proteins [Fesik, 2000; Huang & Strasser, 2000].

In healthy cells, Bak is already inserted into the outer mitochondrial membrane, whereas Bax is a cytosolic protein, with the capacity for translocation to the mitochondrial and ER (endoplasmic reticulum) membranes upon activation. According to the current dogma, even activated proapoptotic effectors Bax and Bak can be sequestrated and neutralized by the prosurvival Bcl‐2 members [Kroemer et al., 2007]. Upon reception of an apoptotic signal, one or more BH3‐only proteins undergo transcriptional or post‐transcriptional activation. Activated BH3‐only proteins either antagonize the prosurvival Bcl‐2 members (‘sensitizers’ or ‘de‐repressors’, e.g., Noxa, Puma, Bad) or also directly act on the proapoptotic effectors (‘direct activators’, e.g., Bid and Bim), resulting in freeing Bax or Bak. The last two undergo conformational changes and/or insertion (Bax) into the outer mitochondrial membrane followed by oligomerization. This leads to MOMP (mitochondrial outer membrane permeabilization), which is the key event in the intrinsic apoptotic pathway [Kroemer et al., 2007; Chipuk & Green, 2008]. As a result, apoptogenic factors, such as cytochrome *c*, become released from the mitochondria triggering a downstream cascade of events, including caspase activation [Danial & Korsmeyer, 2004].

**Figure 1:**
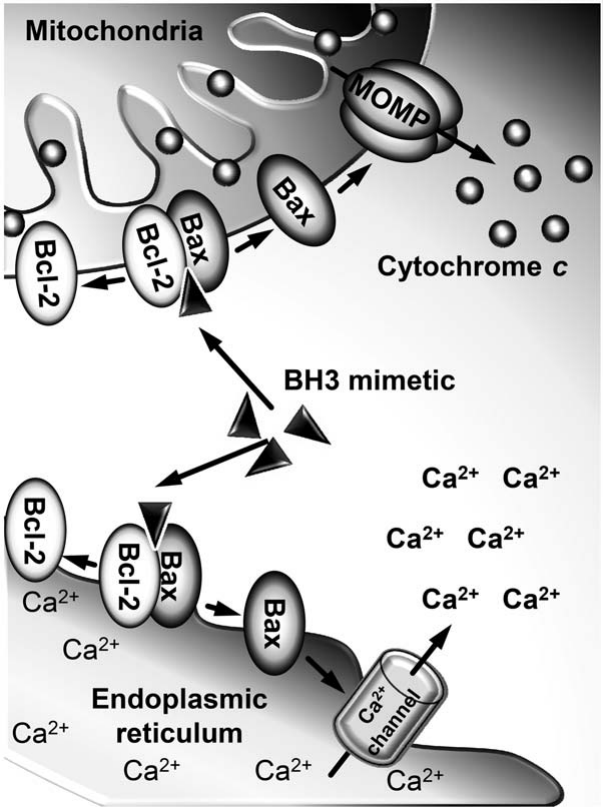
Schematic illustration of the intracellular effects of BH3 mimetics. BH3 mimetics disrupt the heterodimerization of the prosurvival (e.g. Bcl‐2) and pro‐apoptotic (e.g. Bax) Bcl‐2 members located at different intracellular compartments such as the endoplasmic reticulum or mitochondria. Liberation of the proapoptotic proteins leads to (1) the formation of MOMP (mitochondrial outer membrane permeabilization) followed by the release of cytochrome *c* from mitochondria, (2) as well as the release of Ca^2+^ from the intracellular stores.

Given that increased levels of Bcl‐2 proteins have been reported in different cancer types correlating with chemotherapy resistance and poor prognosis [Miyashita & Reed, 1993], Bcl‐2 proteins have become a viable target for anticancer therapy. Substantial efforts in this field yielded in development of synthetic compounds binding to the hydrophobic cleft of the pro‐survival Bcl‐2 proteins such as Bcl‐2 and Bcl‐xL, which results in the inhibition of heterodimerization of the prosurvival and proapoptotic Bcl‐2 family members. This leads to the release and activation of Bax and Bak, followed by induction of apoptosis (Fig. 1). Those largely terphenyl‐based compounds have been termed BH3 mimetics as they reproduce the spatial arrangement of key amino acids in the BH3 domain. In contrast to their prototypes, BH3 peptides, BH3 mimetics are characterized by better stability and therefore have a greater therapeutic potential for controlled inhibition of the pro‐survival Bcl‐2 members [Lessene et al., 2008].

### BH3 MIMETICS

The first BH3 mimetic obtained by molecular modeling and computer screening, HA14‐1, was able to displace Bax from Bcl‐2 and induce apoptosis *in vitro*, characterized by loss of mitochondrial potential and activation of caspases [Wang et al., 2000]. Soon after, two structurally unrelated groups of BH3 inhibitors (BH3Is), derived from BH3I‐1 and BH3I‐2, were discovered in a fluorescence polarization‐based screening [Degterev et al., 2001]. BH3Is were found to displace Bak peptide from Bcl‐xL and induce apoptosis characterized by cytochrome *c* release and caspase activation [Degterev et al., 2001]. In the meantime, the anticancer effects of gossypol isolated from the cotton plant (*Gossypium*) have been attributed to inhibition of Bcl‐2, Bcl‐xL, and Mcl‐1 [Kitada et al., 2003]. In 2005, ABT‐737 was developed [Oltersdorf et al., 2005]. This small‐molecule inhibitor of Bcl‐2, Bcl‐xL, and Bcl‐w, was two‐three orders of magnitude more potent than the previous BH3 mimetics. It did not induce apoptosis on its own, but rather sensitized cells to cell death signals, demonstrating efficacy with chemotherapeutic agents and radiation [Oltersdorf et al., 2005]. The oral bioavailability of this agent was improved even further, resulting in ABT‐263 (Navitoclax), a Bad‐like BH3 mimetic, capable of triggering Bax translocation, cytochrome *c* release, and subsequent apoptosis [Tse et al., 2008]. However, both ABT‐737 and ABT‐263 were found to induce thrombocytopenia and transient thrombocytopathy that severely hindered their therapeutic use [Schoenwaelder et al., 2011]. Recently, Navitoclax was re‐engineered to create a potent, orally bioavailable inhibitor selective for Bcl‐2, ABT‐199 (Venetoclax) [Souers et al., 2013], which has become the first clinically approved small molecule targeting a protein–protein interaction for treating CLL (chronic lymphocytic leukemia) [Green, 2016]. On‐going clinical trials using BH3 mimetics are listed in Table 1.

## CALCIUM SIGNALING

Ca^2+^ signaling is one of the most important types of intracellular communication implicated in a wide variety of biological processes, including cell proliferation [Borowiec et al., 2014], migration [Wei et al., 2012], adhesion [Sheng et al., 2013], fertilization [Armant, 2015], muscle contraction [Bers, 2002], neuronal physiology and signal transmission [Brini et al., 2014], exocytosis [Petersen, 1992] and cell death [Criddle et al., 2007]. Therefore, it is not at all surprising that in the past two decades substantial evidence has accumulated for the role of Bcl‐2 proteins in the regulation of multiple aspects of the intracellular Ca^2+^ homeostasis [Vervliet et al., 2016]. These proteins have been found not only at the mitochondrial membranes, but are also present in the cytosol, at the nuclear envelope as well as at the ER, the main intracellular Ca^2+^ store [Akao et al., 1994]. They directly interact with Ca^2+^ channels and pumps affecting Ca^2+^ release and the steady state ER Ca^2+^ levels. For example, depending on the site of interaction, Bcl‐2 can act either as a direct inhibitor or sensitizer of endoplasmic IP_3_Rs (inositol triphosphate receptors) [Rong et al., 2009; Monaco et al., 2012]. The sensitizing effect is also shared by Bcl‐xL and Mcl‐1 [White et al., 2005; Eckenrode et al., 2010]. Further, Bcl‐2 and Bcl‐xL can directly bind to RyRs (ryanodine receptors) and inhibit RyR‐mediated Ca^2+^ release from the ER [Vervliet et al., 2014; Vervliet et al., 2015]. Bcl‐2 may either protect the function of SERCA (sarco/endoplasmic reticulum Ca^2+^‐ATPase) [He et al., 1997], or destabilize it [Dremina et al., 2006]. At the mitochondrial membranes, Bcl‐2 and Bcl‐xL have been demonstrated to directly inhibit mitochondrial Ca^2+^ uptake via VDAC1 (voltage‐dependent anion channel 1), a large conductance channel permeable to ions and metabolites [Arbel and Shoshan‐Barmatz, 2010; Arbel et al., 2012]; whereas Mcl‐1 was shown to have the opposite effect [H. Huang et al., 2014]. Bcl‐2 may also inhibit mitochondrial NCX (Na^+^/Ca^2+^ exchanger), increasing Ca^2+^ retention in this organelle [Zhu et al., 2001]. Finally, Bcl‐2 can suppress PMCA (plasma membrane Ca^2+^‐ATPase)‐mediated Ca^2+^ extrusion with important implications for cell fate [Ferdek et al., 2012].

## BH3 MIMETICS AND CALCIUM

Given the above, it might be expected that pharmacological inhibition of the pro‐survival Bcl‐2 proteins by BH3 mimetics could, in principle, affect the intracellular Ca^2+^ homeostasis. Indeed, the research has demonstrated that the early mimetics, HA14‐1 and BH3I‐2′, caused a slow and complete release of Ca^2+^ from the ER, followed by a sustained elevation of cytosolic Ca^2+^ concentration in pancreatic acinar cells [Gerasimenko et al., 2010]. Although this effect might be beneficial in cancer, in healthy cells Ca^2+^ overload is undesirable as it promotes cell death, particularly necrosis [Criddle et al., 2007]. This Ca^2+^ release was shown to be attenuated, but not completely blocked, by inhibition of IP_3_Rs and RyRs as well as substantially reduced by strong intracellular Ca^2+^ buffering. Importantly, inhibition of IP_3_Rs and RyRs dramatically reduced BH3I‐2′‐elicited apoptosis, indicating that Ca^2+^ release from the ER contributed to cell death induction by this BH3 mimetic [Gerasimenko et al., 2010]. Similar effects of Ca^2+^ deregulation by HA14–1 were also demonstrated in platelets, HeLa and HEK‐293T cells [Akl et al., 2013]. A recent study has shed new light on this phenomenon by showing that Ca^2+^ responses induced in pancreatic acinar cells by HA14–1, BH3I‐2′ and gossypol were largely diminished in the absence of Bax, but not Bak or Bcl‐2 [Ferdek et al., 2017], suggesting a regulatory role for Bax in Ca^2+^ release from the intracellular stores (Fig. 1). Of note is that BH3 mimetics in this study caused not only apoptosis, but also substantial levels of necrosis in pancreatic acinar cells, both of which were inhibited by strong Ca^2+^ buffering, again pointing towards a Ca^2+^‐dependent component in the mechanism of BH3 mimetic‐induced killing. Since global and sustained Ca^2+^ signals are associated with induction of necrosis, fine tuning of these signals could be useful in shifting unfavorable necrosis towards more physiological apoptosis and thus limiting the side effects of a BH3 mimetic therapy. This has been achieved by CALPs (Ca^2+^‐like peptides), which, by binding to the EF‐hand motifs, mimic the effects of Ca^2+^, pre‐activating various Ca^2+^‐sensitive intracellular targets such as calmodulin and Ca^2+^ channels and pumps [Villain et al., 2000]. CALPs partially reduced Ca^2+^ responses induced by BH3 mimetics resulting in necrosis inhibition or a significant shift in cell death towards apoptosis [Ferdek et al., 2017]. This demonstrates that even a nonspecific inhibition of intracellular Ca^2+^ fluxes can attenuate pathophysiological Ca^2+^ responses and influence the cell death mode and thus may improve the outcome of anticancer therapies.

It is worth noting that not all BH3 mimetics can affect Ca^2+^ homeostasis. A few studies were unable to demonstrate any significant Ca^2+^ release induced by ABT‐737 in platelets and cell lines [Schoenwaelder & Jackson, 2012; Akl et al., 2013] or by ABT‐199 in various *in vitro* models [Vervloessem et al., 2017]. It remains unclear why some BH3 mimetics trigger Ca^2+^ release from the intracellular stores, whereas others do not share this effect. Given the strong dependence of Ca^2+^ responses on the presence of Bax, it is rather unlikely that off‐target effects of early BH3 mimetics are entirely responsible for this phenomenon.

## CONCLUSION

In conclusion, extensive research on inhibitors of the prosurvival Bcl‐2 members yielded a new class of anticancer agents, showing promise particularly against leukemia and lymphoma. Initial excitement, however, slightly faded when the early compounds showed marked side effects. Some of these effects have been attributed to deregulated intracellular Ca^2+^ homeostasis. Despite that, the efforts continued to tailor the specificity of BH3 mimetics in order to preserve the anticancer activity and reduce the undesirable effects. This resulted in ABT‐199, the first clinically approved drug targeting a protein–protein interaction [Green, 2016]. Current clinical trials attempt to combine BH3 mimetics with existing chemotherapeutic agents (Table 1). Nevertheless, it might become essential to establish whether any new BH3 mimetic deregulates intracellular Ca^2+^ release in healthy cells. What is more, in order to increase the safety and efficacy of BH3 mimetic drugs, simultaneous application of agents that regulate intracellular Ca^2+^ homeostasis might be taken into consideration.

**Table 1:** Clinical Trials on BH3 Mimetics (https://clinicaltrials.gov)

BH3‐mimetic	Protein	Disease target	Active clinical trial stage			Estimated completion
(Alternative name)	target	(Additional agent)	I	II	III	
**ABT‐199**	Bcl‐2	AML (Cytarabine)	+			2019
(Venetoclax*,**)		AML (Cobimetinib or Idasanutlin)	+	+		2019
		AML (Azacitidine or Decitabine)	+			2020
		AML (Azacitidine)			+	2022
		Amyloid light chain amyloidosis (Dexamethasone)	+			2021
		B‐cell lymphoma (Ibrutinib and Rituximab)	+			2020
		B‐cell lymphoma (Obinutuzumab)		+		2020
		B‐cell N‐HL (Lenalidomide and Obinutuzumab)	+			2021
		CLL (Bendamustine and Obinutuzumab or Bendamustine and Rituximab)	+			2020
		CLL or SLL (Ibrutinib)	+	+		2021
		CLL (Ibrutinib and Obinutuzumab)	+	+		N/A
		CLL (Allopurinol and Ibrutinib)		+		2022
		CLL (–)			+	2022
		CLL (multiple)		+		2023
		CLL (–)		+		2024
		CLL or SLL (Ibrutinib)		+		2024
		Expanded access program for AML, CLL, MM, N‐HL (–)				N/A
		FL (Obinutuzumab)	+			2020
		FL (Ibrutinib)	+	+		2021
		FL (Obinutuzumab and Polatuzumab Vedotin)	+	+		2021
		MDS (Azacitidine)		+		2019
		MDS (Azacitidine)	+			2020
		MM (Bortezomib and Dexamethasone)			+	2020
		MM (multiple)	+			2021
		MM (Carfilzomib and Dexamethasone)		+		2021
		N‐HL (Ibrutinib)		+		2018
		N‐HL (multiple)	+	+		2019
		N‐HL (–)	+	+		2019
		Waldenstrom macroglobulinemia (–)		+		2023
**ABT‐263**	Bcl‐2	Advanced or metastatic solid tumors (Trametinib)	+	+		N/A
(Navitoclax)	Bcl‐xL	CLL or N‐HL (Rituximab)	+			2018
	Bcl‐w	CLL (–)		+		2018
		Melanoma or solid tumors (Dabrafenib or Trametinib)	+	+		N/A
		Non‐small cell lung carcinoma (Osimertinib)	+			N/A
		Ovarian cancer (–)		+		2018
**AT‐101**	Bcl‐2	CLL (Lenalidomide)	+	+		2018
(R‐(‐)‐Gossypol acetic acid)	Bcl‐xL	Laryngeal cancer (multiple)		+		2018
	Mcl‐1	MM (Dexamethasone and Lenalidomide)	+	+		2021
**PNT2258**	Bcl‐2	B‐cell lymphoma (–)		+		2018
**S 055746**	Bcl‐2	AML or MDS (–)	+			2018

AML acute myeloid leukemia; CLL chronic lymphocytic leukemia; FL follicular lymphoma; MDS myelodysplastic syndromes; MM multiple myeloma; N/A not available on May 29, 2017; N‐HL Non‐Hodgking lymphoma; SLL small lymphocytic lymphoma.

^*^New drug Venxlexta for CLL in patients with a specific chromosomal abnormality ^**^, approved by The US Food and Drug Administration on April 11, 2016

^**^an orphan drug designation.
